# Hyponatremia and aging-related diseases: key player or innocent bystander? A systematic review

**DOI:** 10.1186/s13643-023-02246-w

**Published:** 2023-05-13

**Authors:** Luigia Fratangelo, Sylvain Nguyen, Patrizia D’Amelio

**Affiliations:** 1 Service of Geriatric Medicine & Geriatric Rehabilitation, Department of Medicine, Lausanne University Hospital and University of Lausanne, Lausanne, Switzerland; 2 Department of Medical Science, Geriatric Unit, University of Torino, 10126 Turin, Italy

**Keywords:** Aging, Hyponatremia, Falls, Dementia, Osteoporosis, Fractures

## Abstract

**Background:**

Hyponatremia is frequent in older age; whether it is a key player, a surrogate marker, or an innocent bystander in age-related diseases is still unclear.

*Objective*: To understand the role of hyponatremia in falls, osteoporosis, fractures, and cognitive impairment in old patients.

**Method:**

Eligibility criteria for study inclusions were: written in English, peer-reviewed observational and intervention studies, clinical trial, prospective and retrospective controlled cohort studies, and case-controlled studies without limitations regarding the date of publication.

*Information sources*: Protocol available on the International Prospective Register of Systematic Reviews (PROSPERO, CRD42021218389). MEDLINE, Embase, and PsycINFO were searched. Final search done on August 8, 2021.

*Risk-of-bias assessment*: Risk-of-Bias Assessment tool for Non-randomized Studies (RoBANS) and the Bradford Hill’s criteria for causality.

**Results:**

*Includes studies*: One-hundred thirty-five articles retained for the revision.

*Synthesis of results* — *Falls*: Eleven studies were included. Strong association between hyponatremia and falls in all the studies was found. *Osteoporosis and fractures*: nineteen articles were included. The association between hyponatremia and osteoporosis is unclear. *Cognitive impairment*: Five articles were included. No association between hyponatremia and cognitive impairment was found.

**Discussion:**

*Interpretation*: Falls, osteoporosis, and fractures are multifactorial. Hyponatremia is not temporally related with the outcomes; we suggest that hyponatremia may be regarded as a marker of unhealthy aging and a confounder instead of a causal factor or an innocent bystander for falls and fractures. Concerning cognitive impairment, there are no evidence supporting a real role of hyponatremia to be regarded as an innocent bystander in neurodegeneration.

## Introduction

During the last century, the life expectancy has doubled; however, the increase in “healthy life expectancy” has not increased superimposably [1, 2]. Many factors influence aging as follows: geriatrics syndromes, such as impaired mobility, incontinence, cognitive impairment, dementia, but also environmental and social factors, can affect health maintenance. A healthy lifestyle comprised of varied and balanced nutrition and regular physical activity; abstention from smoking and alcohol contributes to healthy aging by maintaining independence in daily activities and cognition [3]. Unhealthy or frail aging is characterized by reduced homeostasis and increased risk of poor health outcomes. Early identification of the risk factors leading to “healthy” or “unhealthy” aging is fundamental to suggest preventive measures and early treatment. Amongst factors associated with unhealthy aging, hyponatremia has been identified as a possible determinant of comorbidity and poor quality of life. Hyponatremia is the most common electrolytic disorder amongst adults aged 65 years and older; it is defined as a serum sodium concentration lower than 135 mEq/l and indicates a relative excess of water compared to sodium, as the homeostasis of water is the main determinant of the plasma concentration of this cation [4]. Prevalence widely varies depending on different clinical settings ranging from 7.5 to 11% amongst older community-dwelling subjects, whereas it can occur in up to 53% in nursing home residents [5, 6].

Amongst older subjects, the syndrome of inappropriate antidiuretic hormone secretion (SIADH) is responsible of 50% of chronic hyponatremia; however, hyponatremia may be associated to the use of drugs such as diuretics, selective serotonin reuptake inhibitors (SSRIs) and antidepressants, and diseases as liver cirrhosis, nephropathies, adrenal insufficiency, congestive heart failure, and hypothyroidism [7]. In older subjects, hyponatremia is often chronic and rather mild with a serum sodium between 130 and 135 mEq/l [8].

While severe acute hyponatremia is a well-known cause of neurological symptoms due to cerebral edema, older adults with mild and moderate hyponatremia do not develop specific symptoms; thus, the diagnosis is often missed or delayed. Increasing evidences suggest that hyponatremia may be associated with poor clinical outcomes in older subjects including falls [9], osteoporosis [10], fractures [11], neurocognitive disorders [12], and increased morbidity and mortality [13]. However, it is not clear if hyponatremia plays a causal role in these conditions or may be rather regarded as a surrogate marker of poor health and unhealthy aging.

The association between hyponatremia and increased risk of falls has been explained mainly by the impaired balance and the attention deficit associated to this electrolytic disorder [9, 14]. This has been further supported by experimental models showing that hyponatremia causes gait abnormalities via the increase in extracellular glutamate concentration due to astrocytic glutamate decreased uptake [15]. Beside the effects of hyponatremia, some drugs associated with reduced serum sodium (e.g., SSRIs) may cause sensory and motor deficits increasing the risk of falls; in fact, these drugs have been associated to falls and fractures even in the absence of hyponatremia [16].

Hyponatremia has also been associated with increased fracture risk; besides the increased risk of falls [8, 10], hyponatremia may favor bone resorption and osteoporosis, which has been clearly demonstrated in animal models [7, 17, 18]. In humans, however, association studies do not fully support a direct effect of hyponatremia on bone density [4, 19, 20], and hyponatremia has rather been interpreted as general marker of poor health status than an independent risk of fractures [21].

Regarding cognitive impairment, data are sparse in humans [9, 13]; hyponatremic older subjects have worse cognitive and functional performance as respect to non-hyponatremic age-matched controls [8, 13]. A possible explanation may be the reduced synthesis glutamate, demonstrated in animal models [22].

Some studies focused on the association between increased mortality and hyponatremia [4, 10, 21, 23, 24], suggesting that the latter may be considered as a general marker of poor health status rather than as a causal factor of unhealthy aging.

The aim of this systematic review is to explore whether hyponatremia may be considered rather a key player, a surrogate marker, or an innocent bystander in the occurrence of falls, fractures, and cognitive impairment.

## Materials and methods

### Eligibility criteria

Studies included in this systematic review answered the research question structured by the following PI/ECO (participants, intervention/exposure, comparator, outcomes) format.

- *Participants*: Older adults defined as 65 years of age and older
- *Interventions/exposure*: Hyponatremia
- *Comparator*: Normal sodium levels
- *Outcomes*: Falls, osteoporosis with or without fractures, dementia.
- *Study design*: We included English-language peer-reviewed observational and intervention studies, clinical trial, prospective and retrospective controlled cohort studies, and case-controlled studies without limitations regarding the date of publication. Only studies in humans have been reviewed.

### Information source and search strategy

We carried out this systematic review in agreement with the Preferred Reporting Items for Systematic Reviews and Meta-Analysis (PRISMA). The protocol of this study is available on the International Prospective Register of Systematic Reviews (PROSPERO, number CRD42021218389, https://www.crd.york.ac.uk/PROSPERO/display_record.php?RecordID=218389). The MEDLINE, Embase, and PsycINFO database were searched for relevant studies using the following terms: (("Aged"[Mesh] OR "Frail Elderly"[Mesh]) AND "Hyponatremia"[Mesh] AND ("Accidental Falls"[Mesh] OR ("Dementia"[Mesh] OR "Frontotemporal Dementia"[Mesh] OR "Mental Status and Dementia Tests"[Mesh]) OR "Osteoporosis"[Mesh] OR ("Fractures, Bone"[Mesh] OR "Fractures, Spontaneous"[Mesh] OR "Osteoporotic Fractures"[Mesh]). The search strategy is publicly available at https://PubMed.ncbi.nlm.nih.gov/?term=%28%28%22Aged%22%5BMesh%5D+OR+%22Frail+Elderly%22%5BMesh%5D%29+AND+%22Hyponatremia%22%5BMesh%5D+AND+%28%22Accidental+Falls%22%5BMesh%5D+OR+%28%22Dementia%22%5BMesh%5D+OR+%22Frontotemporal+Dementia%22%5BMesh%5D+OR+%22Mental+Status+and+Dementia+Tests%22%5BMesh%5D+%29+OR+%22Osteoporosis%22%5BMesh%5D+OR+%28%22Fractures%2C+Bone%22%5BMesh%5D+OR+%22Fractures%2C+Spontaneous%22%5BMesh%5D+OR+%22Osteoporotic+Fractures%22%5BMesh%5D%29%29%29&filter=hum_ani.humans&filter=lang.english&filter=age.80andover&filter=age.aged&sort=date.

### Study selection

Three reviewers working independently identified studies meeting criteria for inclusion and check decisions. Two reviewers independently evaluated each study; discrepancies between the two reviewers were solved by the third. The final search was done on August 8, 2021. All the papers retrieved by the search responding to inclusion/exclusion criteria were included; biases were evaluated for each article and noted in a developed database. In order to evaluate bias, we used the Risk of Bias Assessment tool for Non-randomized Studies (RoBANS) [25].

### Data extraction

From each study, we extracted the following: publication year, design of the study, participants number, number of hyponatremic and non-hyponatremic patient, definition of hyponatremia, timing of sodium measurement, mean serum sodium, etiology of hyponatremia, use of diuretics, mean age, gender, comorbidities and characteristic of participants, main outcome, scores, and methods used to define the outcomes and results.

We used the Hill’s criteria for causality [26] to verify the existence of a causal relationship between hyponatremia and outcomes. Accordingly, we considered the following associative aspects: strength, consistency, specificity, temporality, biological gradient, plausibility, coherence, experiment, and analogy. In the analysis reported below, these criteria are examined for each paper of this systematic review to clarify whether hyponatremia is a causal factor (key player), if it is a risk factor (marker), or whether it has no association with the considered outcomes (bystander).

### Data analysis

We have first analyzed whether a meta-analysis was possible according to the elements listed in the Sect. 12.1 of the *Cochrane Handbook for Systematic Reviews of Interventions* v6.3 [27]. In this respect, we identified the following issues: (i) across the identified studies, the considered outcomes (i.e., falls, osteoporosis with or without fractures, dementia) in relation to hyponatremia have been treated differently in view of the different time-to-event outcome; furthermore, the definition of hyponatremia threshold was different (see Tables 1, 2, and 3 described in the “Results” Sect. 3); (ii) the design of the studies was also heterogeneous.

**Table 1:** Hyponatremia and falls: characteristics of the studies included in the review

Study	Study design	Sodium level	Definition of hyponatremia used	Timing of sodium measurement	Participants (number)	Age of participants (years)	Gender	Etiology of hyponatremia	Scores	Diuretics	Main outcomes
Renneboog et al. (2006) [9]	Case control	125 ± 5 mEq/l (mean ± SD) in hyponatremia group	Serum sodium < 132 mEq/l	At admission and after 72-h intervals	366	70 (14) Mean (SD)	56.56% F	SIADH, diuretic-induced hyponatremia, salt depletion, polydipsia-hyponatremia syndrome, tubulopathy, transient SIADH	TTW	Type and dosage of diuretic not specified	Incidence of falls in MED
Hoorn et al. (2011) [4]	Prospective cohort	133.4 ± 2.0 mEq/l (total mean ± SD)	Serum sodium < 136 mEq/l	Single at baseline	5208	70.3 (9.1) Mean (SD)	61.5% F	Not evaluated	-	Thiazides, loop diuretics, potassium-sparing diuretics. Dosage not reported	Incidence of fractures, falls, mortality in the community
Gosch et al. (2012) [13]	Case control	127.98 mEq/l (mean) in hyponatremia group	Serum sodium < 135 mEq/l	Single at admission	2880	78.6 (6.98) Mean (SD)	75.6% F	Hypo- (4.7%), and normo-osmolar (1.6%) hyponatremia, adverse drug reaction (15.5%), hyponatremia related to heart failure (3.1%), severe liver disease (0.8%), cancer (1.6%), adrenal insufficiency (1.6%), SIADH (0.8%)	CCI, CIRS, ADL, TMT, TUG	Type and dosage of diuretic not specified	Effect of hyponatremia on CGA in patients admitted to the GEMU
Ahamed et al. (2014) [28]	Case control	Not reported	Serum sodium ≤ 134 mE/l	Single at admission	486	80.8 (7.63) Mean (SD)	59.3% F	Not evaluated	CCI	Type and dosage of diuretic not specified	Incidence of falls in hyponatremic patients admitted under GIMU
Ganguli et al. (2015) [29]	Retrospective chart review	131.2 ± 4.5 mEq/l (initial hyponatremia) 130.4 ± 3.5 mEq/l (persistent hyponatremia)	Serum sodium < 136 mEq/l	At baseline, at least 2 or more than 6 consecutive measurements	608	84.3 (9.3) Mean (SD)	77.1% F	Euvolemic (69.8%): main causes were thiazides and SSRI use, idiopathic SIADH; hypervolemic (9.4%): main cause was end-stage renal disease and congestive heart failure; hypovolemic (20.7%): main causes were diarrhea and diuretics (furosemide and thiazide)	CCI	Thiazides, furosemide. Dosage not reported	Incidence of falls, fractures dues to falls, hospitalization, mortality in community-dwelling elderly
Rittenhouse et al. (2015) [30]	Cross-sectional	138 mEq/l (total median)	Serum sodium < 135 mEq/l	Single at admission	2370	80 (74–86) Median (IQR)	60.1% F	Not evaluated	-	Not evaluated	Prevalence of hyponatremia in fallers and mortality in patient admitted to level 2 geriatric trauma center
Tachi et al. (2015) [31]	Cross-sectional	132 mEq/l (median) in hyponatremia group	Serum sodium < 135 mEq/l	Single at admission	2948	64.5% aged 65 years old or older	41.1% F	Not evaluated	-	Type and dosage of diuretic not specified	Prevalence of hyponatremia in hospitalized patients and effect on the risk of falls
Harianto et al. (2017) [32]	Case control	Not reported	Serum sodium < 134 mEq/l	Single at admission	261	82.85 (7.06) Mean (SD)	41.4% F	Not evaluated	-	Type and dosage of diuretic not specified	Prevalence of hyponatremia and incidence of falls in in-hospital patients
Kuo et al. (2017) [33]	Cross-sectional	130.5 ± 4.1 mEq/l (hyponatremic patients > 65 years old)	Serum sodium < 135 mEq/l	Single at admission	2494	≥ 65 years old (= elderly) 20–64 years old (= adult)	63.2% F	Not evaluated	-	Not evaluated	Prevalence of hyponatremia in fallers, mortality in patients admitted to level 1 trauma center
Hosseini et al. (2018) [34]	Prospective cohort	140 ± 2.3 mEq/l (mean ± SD) in falls group	Serum sodium ≤ 137 mEq/l	Single at baseline	1113	68.6 (6.8) Mean (SD)	44.1%F	Not evaluated	ADL, BBS	Patient under thiazides were excluded. Dosage not reported	Incidence of bone fracture and falls
Boyer et al. (2019) [35]	Cross-sectional	Not reported	Serum sodium < 136 mEq/l	Single at admission	696	86.1 (5.6) Mean (SD)	63.1% F	Not evaluated	CGA, ADL, SEGA	Type and dosage of diuretic not specified	Prevalence of mild chronic hyponatremia in fallers and not fallers admitted to the MUPA unit

*CGA* comprehensive geriatric assessment, *ADL* activity of daily living, *SEGA* frailty score on the Short Emergency Geriatric Assessment, *CCI* Charlson Comorbidities Index, *CIRS* Cumulative Illness Rating Scale, *IQR* interquartile range, *MED* Medical Emergency Department, *GIMU* General Internal Medicine Unit, *ISS* Injury Severity Score, *MUPA* Médecine d’Urgence de la Personne Agée, *GEMU* Geriatric Evaluation and Management Unit, *TMT* Tinetti Mobility Test, *TUG* Timed Up and Go test, *TTW* total travelled way, *BBS* Berg Balance Scale, *SIADH* syndrome of inappropriate hormone secretion

**Table 2:** Hyponatremia, osteoporosis, and fractures: characteristics of the studies included in the review

Study	Study design	Sodium level	Definition of hyponatremia used	Time of sodium measurement	Participants (number)	Age of participants (years)	Gender	Etiology of hyponatremia	Scores	Diuretics	Main outcome
Gankam Kengne et al. (2008) [10]	Case control	131 ± 3 mEq/l (mean ± SD) in hyponatremia group	Serum sodium < 135 mEq/l	Single at admission (pretreatment)	513	81 (8) Mean (SD)	74.1% F	Idiopathic SIADH (35%), diuretics (35%), SSRI (16%), salt depletion (6%), secondary SIADH (4%), potomania (3%), antiepileptic drugs (1%)	-	Type and dosage of diuretic not specified	Prevalence of hyponatremia in fractures
Sandhu et al. (2009) [36]	Cross-sectional	131 ± 2 mEq/l (mean ± SD) in fracture group	Serum sodium < 135 mEq/l	Single at baseline	1609	79.2 (8.2) Mean (SD)	75.3% F	Hyponatremia associated with central nervous system disease, lung disease, thyroid disorders, diuretic, or antidepressant use	-	Type and dosage of diuretic not specified	Incidence of hyponatremia in patient with fracture
Kinsella et al. (2010) [19]	Cross-sectional	140.6 ± 3.0 mEq/l (total mean ± SD)	Serum sodium < 135 mEq/l	Within 1 year before DXA	1408	61 (11) Mean (SD)	100% F	Not evaluated	BMD, DXA	Not evaluated	Incidence of fractures
Chow et al. (2011) [37]	Retrospective cohort	116 ± 7 mEq/l (mean ± SD) in the thiazide-induced group	Serum sodium < 135 mEq/l	Record of hyponatremia	439	76 (9) Mean (SD)	70.4% F	Thiazide-induced hyponatremia	-	Thiazide, dosage not reported	Prevalence of fracture in patients with thiazide-induced hyponatremia
Hoorn et al. (2011) [4]	Prospective cohort	140.2 ± 3.3 mEq/l (total mean ± SD)	Serum sodium < 136 mEq/l	Single at baseline	5208	70.3 (9.1) Mean (SD)	61.5% F	Not evaluated	-	Thiazides, loop diuretics, potassium-sparing diuretics. Dosage not reported	Incidence of fractures, falls, mortality in the community
Tolouian et al. (2012) [38]	Case control	137.4 ± 3.8 mEq/l (mean ± SD) in hyponatremia group	Serum sodium < 135 mEq/l	Single at baseline	293	81.6 (8.4) Mean (SD)	63.1% F	Not evaluated	-	Not evaluated	Prevalence of hyponatremia in hip fracture
Arampatzis et al. (2013) [39]	Cross-sectional	139 ± 4 mEq/l (mean ± SD) in hyponatremia group	Serum sodium < 132 mEq/l	Single at admission	10,823	73 (12) Mean (SD)	63% F	Hyponatremia related to diuretic use	-	Thiazides, loop diuretics, spironolactone, amiloride. Dosages not reported	Prevalence of fractures in hyponatremic (loop diuretics users)
Hagino et al. (2013) [40]	Case control	132 ± 2.3 mEq/l (mean ± SD) in hyponatremia group	Serum sodium < 135 mEq/l	Single at admission	512	86.7 (6.6) Mean (SD)	75.5% F	Hyponatremia related to heart failure, liver failure, and diabetes	-	Not evaluated	Prevalence of hyponatremia at in hip fracture
Afshinnia et al. (2015) [41]	Cross-sectional	140.2 ± 2.3 mEq/l (total mean ± SD)	Serum sodium ≤ 135 mEq/l	Time averaged	24,784	61 (14) Mean (SD)	81.6% F	Hyponatremia related to diuretics use and liver cirrhosis	BMD, DXA	Thiazides, loop diuretics. Dosage not reported	Prevalence of osteoporosis
Ganguli et al. (2015) [29]	Retrospective chart review	131.2 ± 4.5 mEq/l (initial hyponatremia) 130.4 ± 3.5 mEq/l (persistent hyponatremia)	Serum sodium < 136 mEq/l	At baseline, at least 2 or more than 6 consecutive measurements	608	84.3 (9.3) Mean (SD)	77.1% F	Euvolemic (69.8%): main causes were thiazides and SSRI use, idiopathic SIADH; hypervolemic (9.4%): main cause was end-stage renal disease and congestive heart failure; hypovolemic (20.7%): main causes were diarrhea and diuretics (furosemide and thiazide)	-	Thiazides, furosemide. Dosage not reported	Incidence of falls, fractures due to falls, hospitalization, mortality
Holm et al. (2015) [42]	Retrospective cohort	141.5 ± 2.8 mEq/l (total mean ± SD)	Serum sodium < 136 mEq/l	Single at baseline	5610	61.4 (11.7) Mean (SD)	100% F	Hyponatremia related to diuretic use, liver disease, congestive heart disease, diabetes, malignancy	BMD, DXA	Thiazides, loop diuretics, potassium-sparing diuretics. Dosage not reported	Prevalence of osteoporosis
Jamal et al. (2015) [43]	Prospective cohort	132.3 ± 1.8 mEq/l (mean ± SD) in hyponatremia group	Serum sodium < 135 mEq/l	Single at baseline	5122	76.8 (7.0) Mean (SD)	100% M	Hyponatremia related to congestive heart failure, diabetes	BMD, DXA	Thiazides, non-thiazides. Dosage not reported	Prevalence of morphometric fractures
Kruse et al. (2015) [20]	Cross-sectional	139.4 ± 3.08 MEq/l (total mean ± SD)	Serum sodium < 135 mEq/l	Within 14 days before or after DXA	1575	63.13 (13.6) Mean (SD)	77.2% F	Hyponatremia related to liver insufficiency, ischemic heart disease, diabetes, malignancy, chronic kidney disease	BMD, DXA	Thiazides, loop diuretics. Dosage not reported	Prevalence of osteoporosis
Usala et al. (2015) [44]	Case control	Not reported	Serum sodium < 135 mEq/l	At least 1 measurement	139,594	65.9 (14.7) Mean (SD)	88.3% F	Not evaluated	ICD-9-CM	Thiazides, loop diuretics. Dosage not reported	Incidence of osteoporosis and fragility fractures
Ayus et al. (2016) [45]	Retrospective cohort	132 ± 5 (mean ± SD) in hyponatremia group	Serum sodium < 135 mEq/l	At least on 2 or more consecutive measurements for > 90 days	31,527	78 (12) Mean (SD)	71.5% F	Hyponatremia related to heart failure, chronic kidney disease, liver failure, diabetes, diuretic use	-	Thiazides. Dosage not reported	Incidence of hip fracture
Hosseini et al. (2018) [34]	Prospective cohort	140 ± 2.3 mEq/l (mean ± SD) in fracture group	Serum sodium ≤ 137 mEq/l	Single at baseline	1113	68.6 (6.8) Mean (SD)	44.1% F	Not available	BMD, DXA	Patient under thiazides was excluded. Dosage not reported	Incidence of bone fracture and falls
Adams et al. (2019) [46]	Retrospective cohort	139 mEq/l (total median)	Serum sodium < 135 mEq/l	Time averaged	341,003	63.3 Median	67% F	Hyponatremia related to cardiovascular disease, diabetes, diuretic use	BMD, DXA	Thiazides. Dosage not reported	Prevalence of osteoporosis
Nigwekar et al. (2019) [47]	Case control	Not reported	Serum sodium < 135 mEq/l	At least two measurements separated by at least 90 days	5751	84 (9) Mean (SD)	61% F	Hyponatremia related to congestive heart failure, cirrhosis, diabetes, diuretic use	-	Type and dosage of diuretic non specified	Prevalence of hyponatremia in hip fracture
Schiara et al. (2020) [21]	Case–control and prospective cohort	129.9 ± 4.7 mEq/l (mean ± SD) in hyponatremia prospective group	Serum sodium < 135 mEq/l	Single at admission	2768 (case control) 284 (cohort)	83 (7) Mean (SD)	77.8% F	Not evaluated	-	Thiazides. Dosage not reported	Prevalence of hyponatremia and hypokalemia, mortality

*BMD* bone mineral density, *DXA* dual-energy X-ray absorptiometry, *ICD-9* International Classification of Disease, Ninth Revision, code 733 for osteoporosis

**Table 3:** Hyponatremia and cognitive impairment: characteristics of the studies included in the review

Study	Study design	Sodium level	Definition of hyponatremia used	Timing of sodium measurement	Participants (number)	Age of participants (years)	Gender	Etiology of hyponatremia	Cognitive scores	Diuretics	Main outcome
Chung et al. (2017) [48]	Retrospective cohort	Not reported	According to ICD-9-CM, no further definition	Single at baseline	24,445	No. of subjects < 65 years: 1797 (36.7%) No. of subjects > 65 years: 3103 (63.3%)	44.8% F	Hyponatremia related to heart failure, liver cirrhosis, diabetes, malignancy, diuretic use	Dementia diagnosed according to ICD-9-CM	Furosemide, thiazides. Dosage not reported	Hyponatremia as a predictor of dementia
C. Fujisawa et al. (2021) [49]	Prospective cohort	Not reported	Serum sodium < 135 mEq/l	Single at baseline	2982	82.0 (76.0–84.0) in hyponatremic vs 79.0 (75–83) in normonatremic Median years (IQR)	48% F	Hyponatremia related to cardiac disease, liver disease, diabetes, diuretic use	MMSE, FAB, Digit span forward, Digit span backward, category fluency, logical memory	Type and dosage of diuretics not specified	Association between hyponatremia and cognitive impairment, muscle mass, physical performance
Gosch et al. (2012) [13]	Case control	128 ± 3.2 mEq/l (mean ± SD) in hyponatremic group	Serum sodium < 135 mEq/l	Single at admission	2880	78.6 (6.98) Mean years (SD)	75.6% F	Hypo- (4.7%), and normo-osmolar (1.6%) hyponatremia, adverse drug reaction (15.5%), hyponatremia related to heart failure (3.1%), severe liver disease (0.8%), cancer (1.6%), adrenal insufficiency (1.6%), SIADH (0.8%)	MMSE, clock completion	Type and dosage of diuretic not specified	Effect of hyponatremia on CGA
Pereira et al. (2006) [50]	Prospective cohort	Not reported	Serum sodium < 135 mEq/l	Single at baseline	306	74 (8.7) Mean years (SD)	50.33% F	Not evaluated	BLAD	Not evaluated	Frequency of laboratory abnormality in MCI and dementia
Suárez et al. (2020) [51]	Prospective cohort	122 mEq/l (median) in hyponatremia group	Serum sodium < 130 mEq/l	Single at admission	180	68 (61–78, cases), 72 (65–78) controls Median years (IQR)	52.31% F	Hyponatremia related to congestive heart disease, liver disease, chronic renal disease, diabetes, malignancy	MMSE, DemTect test, trail-making tests A and B	Thiazides, loop diuretics. Dosage not reported	Differences in cognitive performances between groups, and before and after treatment

*MMSE* mini-mental state examination, *FAB* frontal assessment battery, *CGA* comprehensive geriatric assessment, *TMSE* Thai Mental State Examination, *BLAD* Battery of Lisbon for the Assessment of Dementia, *MCI* mild cognitive impairment, *DemTect* dementia detection

Since statistics (e.g., median, interquartile range) summarizing the effects were available in the identified studies, according to Sect. 12.2.1 of the *Cochrane Handbook for Systematic Reviews of Interventions* v6.3 [27], we opted for the approach “summarizing effect estimates” through the reporting of methods and results. For the sake of transparency, as suggested in [27], we have reported the tabulation of the available effect estimates and discussed them for each outcome.

## Results

### Study selection

**Fig. 1:**
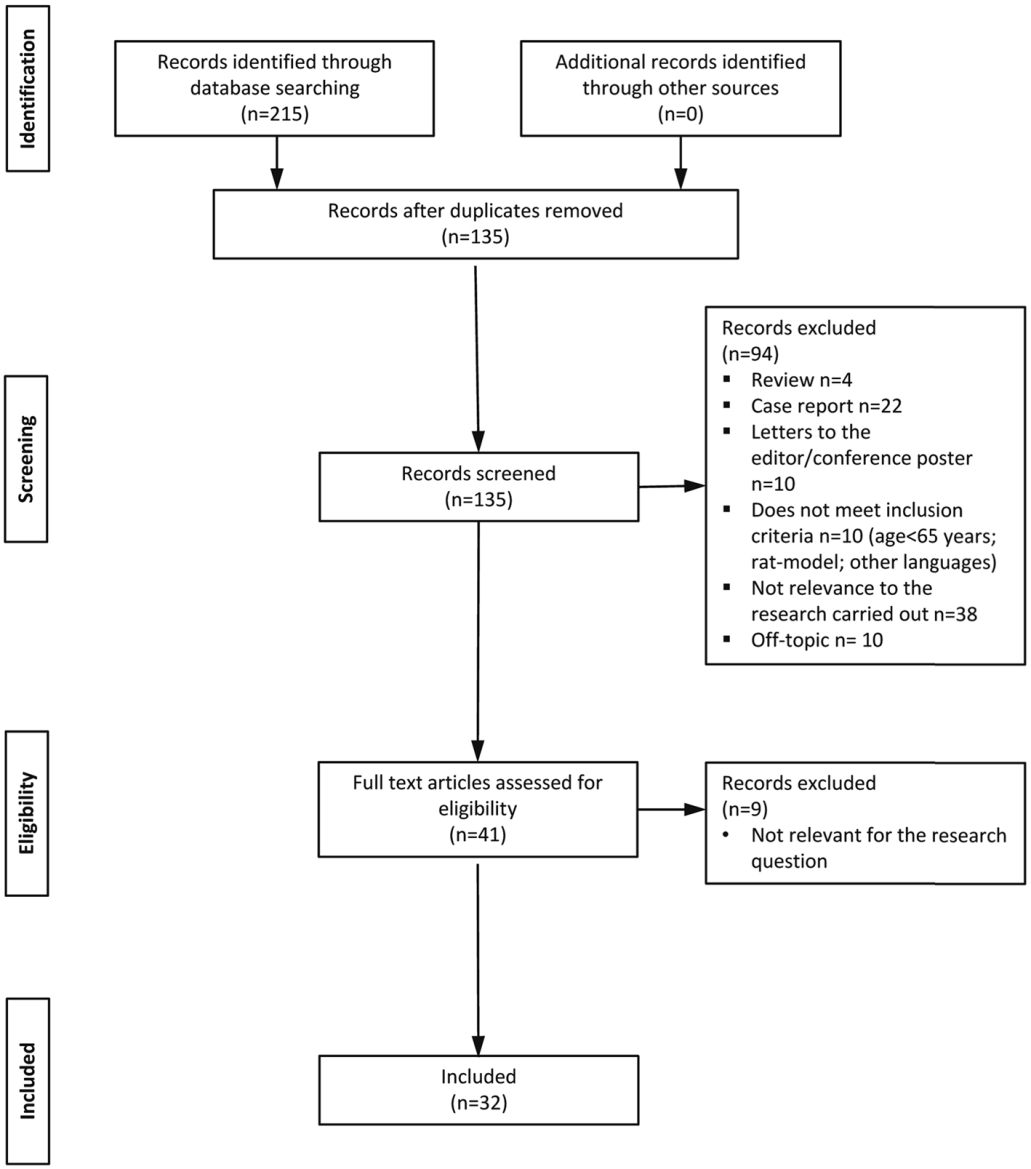
Flow chart of the review procedure

Two-hundred and fifteen articles were retrieved by the search strategy: 107 from PubMed + 61 from Embase + 47 from PsycINFO. After removing the duplicates, we retained 135 articles for the systematic revision. We excluded 94 articles for violation of eligibility criteria; hence, 41 full-text articles were reviewed as previously described. After reading the full-text article, nine articles [52–59] were excluded as non-relevant for the research question (Fig. 1). Papers selected for the review were published between 2002 and 2021. Discrepancies occurred for 3 out of 41 (6.5%) studies and were solved by the third reviewer.

### Hyponatremia and falls

Eleven studies published between 2006 and 2019 were included [4, 9, 13, 28–35], nine of which were retrospective, one a prospective population-based cohort study [4], and one a prospective observational study [34] (Table 1).

Except for the study from Hosseini et al. [34], all the studies evaluated the role of hyponatremia after adjusting for confounders as age, gender, medication, and comorbidities in multivariate analysis.

All the studies used the standard 135 mEq/L in order to define hyponatremia, with the exception of [34] who defined hyponatremic subjects with serum sodium ≤ 137 mEq/L.

An important predictor of falls was being a woman (*OR* 1.62, 95% *CI* 1.32–1.99, *p* < 0.001) [30]. Boyer et al. [35] made a similar observation finding that, amongst fallers, females were older and more dependent than males.

Concerning the studies’ settings, three articles focused on inhospital falls [31, 32, 60]. Amongst those, two found that hyponatremia increases the risk of inhospital falls of 1.8- to 2-folds [31, 32]. However, Harianto et al. [32] found no change in the prevalence of falls according to hyponatremia severity (*p* = 0.267), whereas according to Tachi et al. [31], patients with mild hyponatremia (sodium > 132 mEq/l) did not have an increased risk of falls.

Almost all the studies found that hyponatremia was independently associated with increased risk of falls, with the exception of Hosseini et al. [34] who did not find any difference in the incidence of falls, nor in the static and dynamic balance abilities between hyponatremic and normonatremic patients. According to these authors, these results could be explained by the exclusion from the study of frail patients and of subjects with long-term use of diuretics.

All the studies investigated the role of possible confounders, in particular the burden of comorbidities and functional limitation due to frailty [26]. However, functional impairment and the presence of frailty were assessed with different methods as the Fried Index [29], the frailty score, the Short Emergency Geriatric Assessment and the activity of daily living (ADL) [35], or the sole ADL scale [13]. Comorbidities were assessed using the Charlson Comorbidities Index (CCI) in all the three studies [13, 28, 29].

In several studies, subjects with hyponatremia were older and had increased prevalence of neurological disorder (dementia, history of stroke, Alzheimer’s disease, Parkinson’s disease), of hematologic disorder, and of cardiovascular disease [28, 30, 31, 33]. Despite the increased presence of comorbidity, hyponatremia was associated with falls even after the corrections for confounding factors.

Concerning medications, two studies [4, 28] found a higher prevalence of diuretics use amongst hyponatremic patients: 31.1% in hyponatremic vs 15.0% in controls, *p* < 0.001 [4], and 58% in hyponatremic vs 48.6% in controls, *p* < 0.037 [28]. In the latter, however, fallers exhibited a less prevalent use of diuretic. Furthermore, inhospital falls have been inversely correlated to diuretics as well as with cardiovascular disease [32]. This result has been explained by the adoption of a dedicated multidisciplinary program targeting the risk of falls in the unit concerned.

Almost all the studies analyzed hyponatremia defined from a single measurement, and only one study [29] analyzed possible differences between initial and persistent hyponatremia on the incidence of falls, fractures related to falls, hospitalization, and mortality. Persistent hyponatremia was defined as two or more blood tests showing hyponatremia during a 6-month period.

**Fig. 2:**
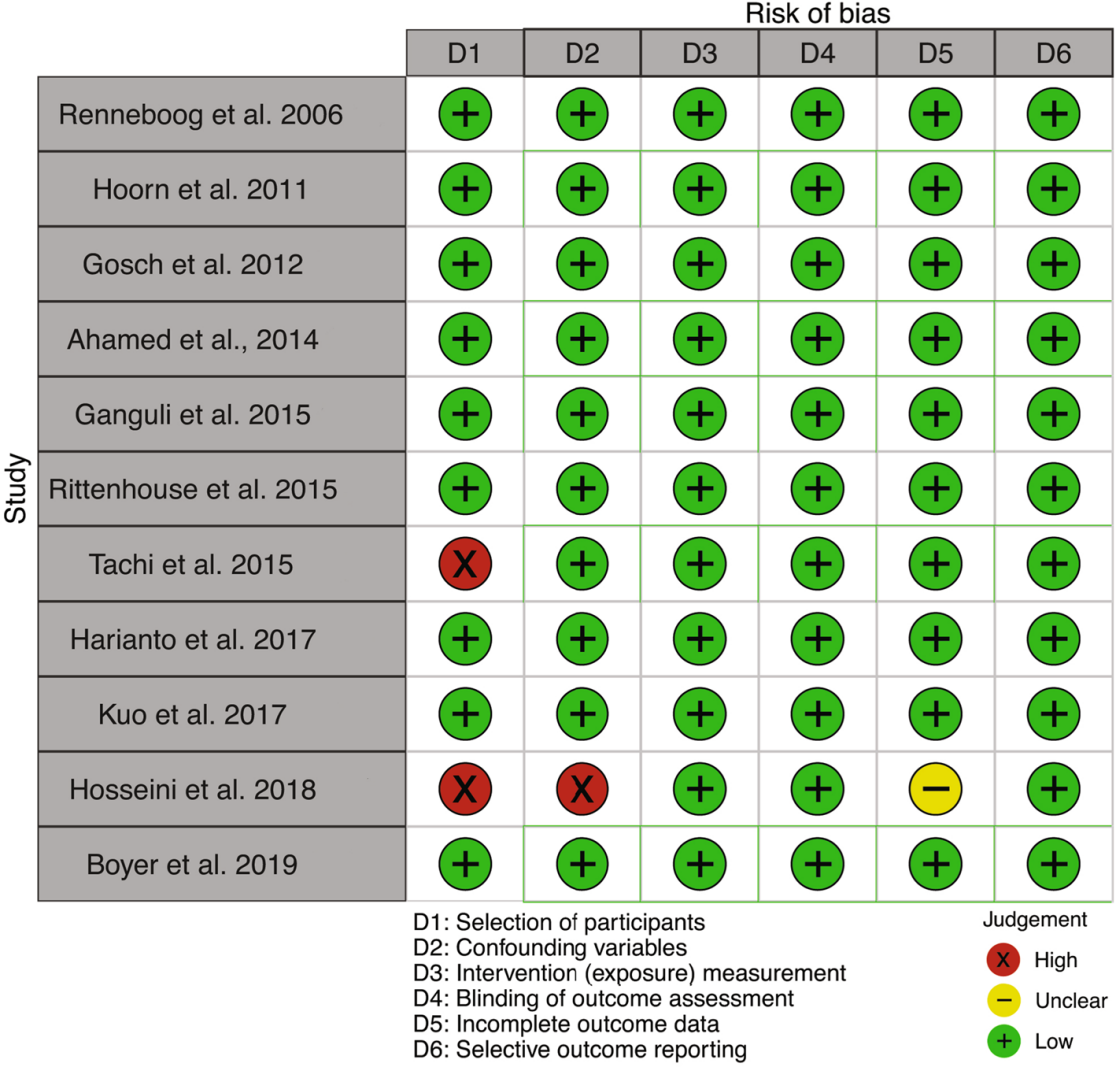
Bias assessment for papers on the relationship between hyponatremia and falls, report for the RoBANS tool

In agreement with the RoBANS criteria, we have estimated that the majority of the studies have a low risk of selection bias with the exception of Tachi et al. [31] due to researcher-dependent definition of a fall used as an exclusion criteria and Hosseini et al. [34] due to exclusion of patients taking thiazides. About confounding bias, in the study of Hosseini et al. [34], comorbidities were not assessed et suitably adjusted. Concerning incomplete outcome data, no information is given about missing data in Hosseini et al. [34]. About the measurement of exposure, blinding of outcome assessment, and selective outcome reporting, all studies have a low risk of bias (Fig. 2).

We analyzed the included studies according to the Bradford Hill’s criteria showing that there is evidence for a strong association between hyponatremia and falls in all the studies (*strength*, Table 4); consistent findings were observed in all the studies (*consistency*); all the studies showed that falls are due to multifactorial causes and hyponatremia may not be regarded as a specific cause (*specificity*); there are no sufficient evidence to temporally relate hyponatremia and falls (*temporality*); and coherence between hyponatremia and falls was demonstrated by all the studies (*coherence*). The criteria referring to the *biological gradient*, the *plausibility*, the presence of *experimental evidences*, and the presence of *analogies* are not applicable to the studies included in this review. However, experimental data confirming the role of hyponatremia in falls were described in previous studies [9, 14, 15].

**Table 4:** Hyponatremia and falls: strength of association according to Bradford Hill’s criteria

Study	Odds ratio (95% *CI*)	*p*-value
Renneboog et al. (2006) [9]	67.43 (7.48–607.42)	0.001
Hoorn et al. (2011) [4]	1.35 (1.03–1.75)	0.029
Gosch et al. (2012) [13]	29.3 (10.90–78.78)	< 0.001
Ahamed et al. (2014) [28]	3.12 (1.84–5.30)	< 0.001
Ganguli et al. (2015) [29]	Not reported	0.0471 (initial hyponatremia) 0.0171 (persistent hyponatremia)
Rittenhouse et al. (2015) [30]	1.81 (1.26–2.60)	0.001
Tachi et al. (2015) [31]	1.751 (1.020–3.005)	< 0.25
Harianto et al. (2017) [32]	Not reported	0.005
Kuo et al. (2017) [33]	2.5 (2.50–3.02)	< 0.001
Hosseini et al. (2018) [34]	1.13 (0.73–1.74)	0.56
Boyer et al. (2019) [35]	3.02 (1.84–4.96)	< 0.001

### Hyponatremia, osteoporosis, and fractures

Nineteen articles published between 2008 and 2020 were included [4, 10, 19–21, 29, 34, 36–47]. Four were prospective cohort studies [4, 21, 34, 43]. The others were retrospective studies [10, 19, 20, 29, 36–47], and one included both a retrospective and prospective part [21]. One study included only men [43] and two only women [19, 42] (Table 2).

Most of the studies considered a single serum sodium measurement at admission or at baseline visit. Some studies measured sodium in the months or years preceding the outcome measure [29, 41, 44–47] since hyponatremia is a condition that persist if uncorrected [41].

Some studies suggested that mild hyponatremia (130 to 134 mEq/l) was associated with fractures owing to a decrease in bone mineral density (BMD) [19, 42, 43]. These results were not confirmed by Hoorn et al. [4] who did not find a lower BMD among older subjects with mild hyponatremia in the large cohort of the Rotterdam study. The authors hypothesized that a severe degree of hyponatremia may be associated to osteoporosis; however, in their study, only 1.5% of subjects were affected by severe hyponatremia. Similar results were found only by Hosseini et al. [34].

The studies focused on persistent hyponatremia [29, 41, 44–47] found an increase in osteoporosis risk in the adjusted models varying from *OR* 1.13–2.12 [41] to *OR* 4.61 [44]. Within the abovementioned studies, osteoporosis was defined as a decrease of bone mineral density (BMD) measured with bone densitometry, according to the standard NHI definition [61].

In cross-sectional studies, the outcome (fracture or reduced BMD) was measured simultaneously with natremia. In both prospective and retrospective studies, the timing of exposure varied between 14 days [20] and 1 year [19]. Only one study calculated a time-weighted mean sodium values considering the time between sodium measurements and diagnosis of osteoporosis [46]. Three studies considered the diagnosis of hyponatremia only if persistent in at least two measurements before the measurement of the outcome [29, 45, 47] (Table 2).

**Fig. 3:**
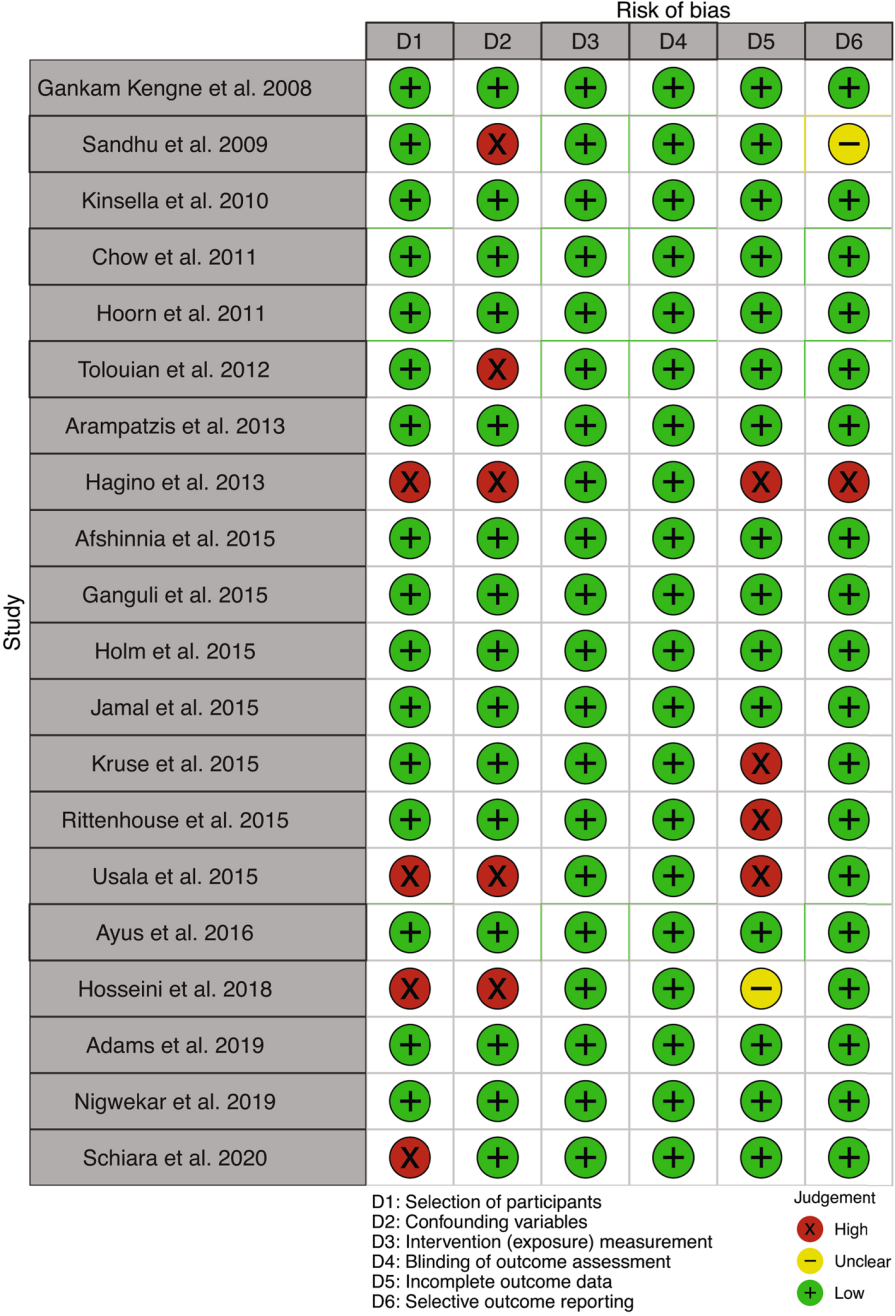
Bias assessment for papers on the relationship between hyponatremia and osteoporosis and/or fractures, report for the RoBANS tool

According to RoBANS criteria, the study of Hagino et al. [40] showed a high risk of selection bias due to an unclear distribution between cases and controls of patients who received conservative treatment. The latter patients could have a higher number of illnesses or more serious diseases that could have affected outcome (mortality) explaining the high risk of confounding bias. In addition, missing data about patients who receive conservative treatment may have created a bias in the survival data and a selective reporting of outcomes. In Usala et al. [44], a matched case–control study, we found a high risk of selection and confounding bias in relation to the study design. Data on patients lost at follow-up were not specified, and this is responsible for an attrition bias (Fig. 3).

The analyses according to the Bradford Hill’s criteria showed that the association between hyponatremia and osteoporosis is unclear (*strength*, Table 5), consistent findings were observed in all the studies (*consistency*); all the studies showed that osteoporosis and falls are due to different causes and hyponatremia may not be regarded as a specific cause (*specificity*); there are no sufficient evidence to temporally relate hyponatremia and osteoporosis and/or fractures (*temporality*); and coherence between hyponatremia and osteoporosis and/or falls was demonstrated by all the studies (*coherence*). The criteria referring to the *biological gradient*, the *plausibility*, the presence of *experimental evidence*, and the presence of *analogies* are not applicable to the studies included in this review. However, experimental data confirming the role of hyponatremia in osteoporosis were described in previous studies [7, 17, 18].

**Table 5:** Hyponatremia and osteoporosis and fractures: strength of association according to Bradford Hill’s criteria

Study	Risk (95% *CI*)	*p*-value
Gankam Kengne et al. (2008) [10]	*OR* 4.16 (2.24–7.71)	< 0.001
Sandhu et al. (2009) [36]	Not reported	0.01
Kinsella et al. (2010) [19]	*OR* 2.25 (1.24–4.09)	0.01
Chow et al. (2011) [37]	*OR* 1.44 (0.77–2.71)	0.26
Hoorn et al. (2011) [4]	*HR* 1.34 (1.08–1.68)	0.009
Tolouian et al. (2012) [38]	*OR* 4.80 (1.06–21.67)	0.04
Arampatzis et al. (2013) [39]	*OR* 1.46 (1.05–2.04)	0.03
Hagino et al. (2013) [40]	Not reported	0.398
Afshinnia et al. (2015) [41]	Not reported	≤ 0.015
Ganguli et al. (2015) [29]	Not reported	0.5513 (initial hyponatremia)
		0.1246 (persistent hyponatremia)
Holm et al. (2015) [42]	*HR* 1.996 (1.096–3.529)	0.022
Jamal et al. (2015) [43]	*HR* 1.67 (1.02–2.69)	Not reported
Kruse et al. (2015) [20]	*OR* 1.516 (0.971–2.37)	0.067
Usala et al. (2015) [44]	Not reported	Not reported
Ayus et al. (2016) [45]	Not reported	Not reported
Hosseini et al. (2018) [34]	*OR* 0.96 (0.71–1.31)	0.83
Adams et al. (2019) [46]	*RR* 1.11 (1.09–1.13)	Not reported
Nigwekar et al. (2019) [47]	*OR* 1.08 (0.89–1.30) in single episode hyponatremia, *OR* 2.65 (2.18–3.22) in chronic prolonged hyponatremia	Not reported
Schiara et al. (2020) [21]	*OR* 2.56 (2.08–3.13) in hyponatremic respect to OA controls, *OR* 0.92 (0.83–1.02) in hyponatremic respect to AMI controls	Not reported

*OR* odds ratio, *RR* relative risk, *HR* hazard ratio, *OA* osteoarthrosis, *AMI* acute myocardial infarction

### Hyponatremia and cognitive impairment

Five articles published between 2006 and 2021 were included [13, 48–51]. Three studies were prospective [49–51] and two retrospective [13, 48] (Table 3). The studies were highly heterogeneous because of different study designs, population included, definition of hyponatremia adopted, different tests used to evaluate cognitive impairment, and different outcomes analyzed.

Regarding the definition of hyponatremia, Chung et al. [48] used the International Classification of Diseases, Ninth Revision, Clinical Modification (ICD-9-CM); hence, they did not give a clear sodium threshold. In all the studies, hyponatremia was solely measured at baseline and associated to cognitive tests performed simultaneously, without any information on previous diagnosis of hyponatremia.

Only one study [49] found a significant difference in the prevalence of hyponatremia according to gender, with a lower percentage of women in the hyponatremic group. 

Scores applied to evaluate cognitive impairment were heterogeneous amongst the studies; mini-mental state examination (MMSE) in three out of five studies is as follows [13, 49, 51]: frontal assessment battery (FAB), Digit span forward, Digit span backward, category fluency, and logical memory were used in [49]; dementia detection test (DemTect) and trail -making tests A and B (TMT-A and TMT-B) were used in [51]; and clock completion test was applied in [13].

Two studies [13, 51] found a reduced MMSE in the hyponatremic subjects. In particular, Gosch et al. [13] reported an MMSE of 26.05 ± 3.64 points in the hyponatremic group and 27.18 ± 3.15 points in normonatremic subjects (*p* = 0.003). In the multivariate analysis, hyponatremia (*OR* 1.96, 95% *CI* 1.05–3.68, *p* = 0.045) and age (*OR* 1.10, 95% *CI* 1.05–1.16, *p* < 0.001) were significant predictors of MMSE lower than 28 [13]. Similarly, Suárez et al. [51] reported a reduced MMSE in hyponatremic patients as compared to normonatremic ones (26.09 ± 4.23 versus 28.74 ± 1.94, *p* < 0.001). Moreover, the authors showed that using a more sensitive test for the diagnosis of mild cognitive impairment (MCI, *i.d.* DemTect vs MMSE), the association between hyponatremia and cognitive impairment was more robust.

On the contrary, Fujisawa et al. [49] showed no differences in cognition between hypo- and normonatremic subjects. These authors extensively assessed cognitive abilities using different tests, namely MMSE, FAB, Digit Span forward, Digit Span backward, fluency subtest of the Hasegawa dementia scale-revised (HDS-R), and memory disorder prevalence.

**Fig. 4:**
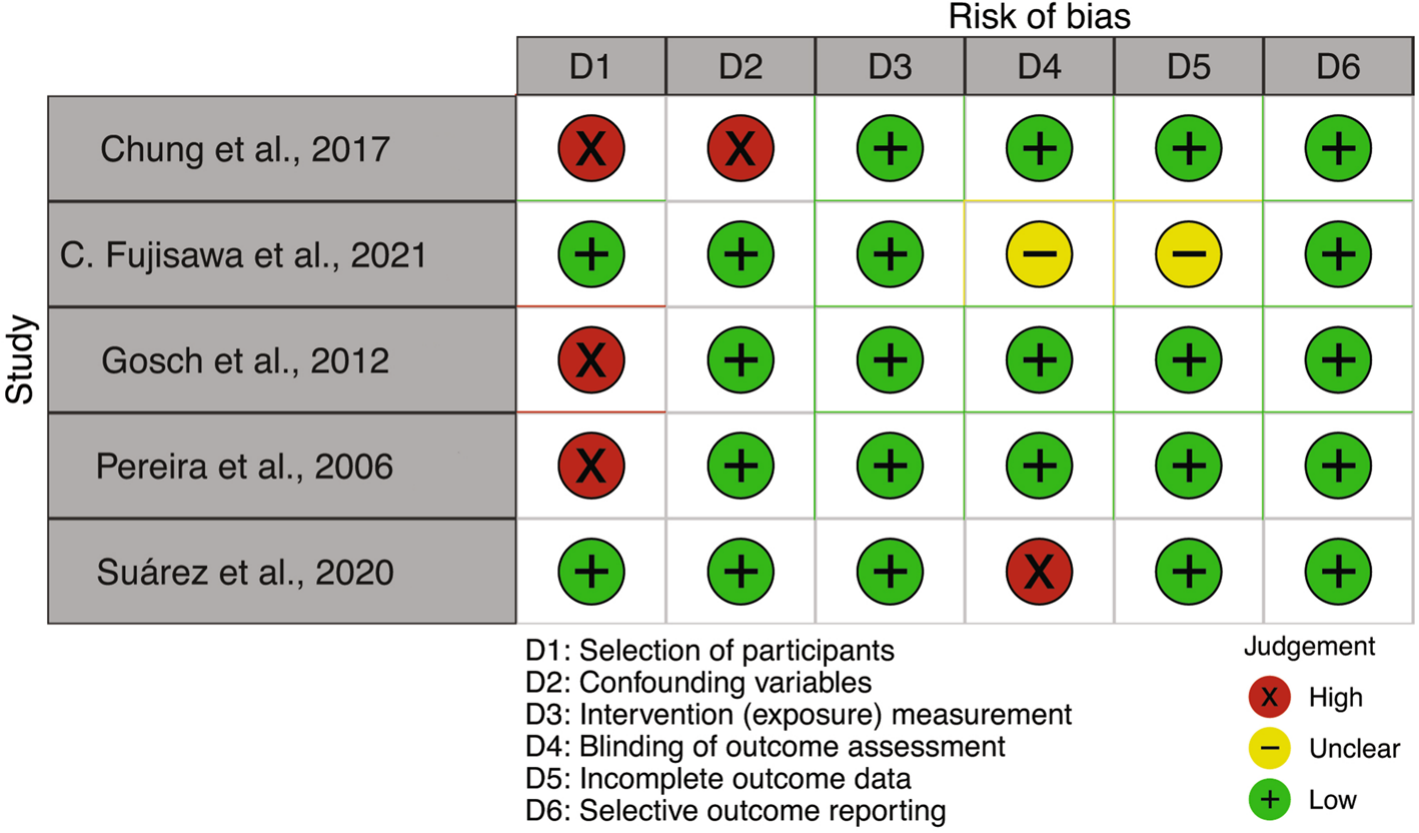
Bias assessment for papers on the relationship between hyponatremia and cognitive impairment, report for the RoBANS tool

According to RoBANS assessment of bias, three out of five studies have a high risk of selection bias. The study of Chung et al. [48], a matched case–control study, shows a selection and confounding bias due to the study design (Fig. 4).

The analyses according to the Bradford Hill’s criteria showed that there are not evidences for an association between hyponatremia and cognitive impairment (*strength*, Table 6); consistent findings were observed in all the studies (*consistency*); all the studies showed that cognitive impairment is multifactorial and hyponatremia may not be regarded as a specific cause (*specificity*); there are no sufficient evidence to temporally relate hyponatremia and cognitive impairment (*temporality*); and coherence between hyponatremia and cognitive impairment was demonstrated by all the studies (*coherence*). The criteria referring to the *biological gradient*, the *plausibility*, the presence of *experimental evidences*, and the presence of *analogies* are not applicable to the studies included in this review. However, experimental data confirming the role of hyponatremia in cognitive impairment were described in previous studies [22].

**Table 6:** Hyponatremia and cognitive impairment: strength of association according to Bradford Hill’s criteria

Study	Relative risk (95% *CI*)	*p*-value
Chung et al. (2017) [48]	*HR* 2.36 (2.09–2.66)	< 0.001
C. Fujisawa et al. (2021) [49]	*OR* 1.1 (0.7–1.9)	0.7
Gosch et al. (2012) [13]	*OR* 1.96 (1.05–4.19)	0.036
Pereira et al. (2006) [50]	Not reported	Not reported
Suárez et al. (2020) [51]	*OR* 3.13 (0.54–25.86) in moderate hyponatremia, *OR* 8.55 (1.63–72.73) in moderate to profound hyponatremia	0.227
		0.022

## Discussion

Hyponatremia is frequent amongst aged subjects and is frequently associated with poor health status. Hence, the question whether it has to be considered a marker of unhealthy aging, an innocent bystander, or rather a cause of disease and unhealthy aging has still not a clear answer. Falls, osteoporosis, fractures, and cognitive impairment burdened the older population and are associated to unhealthy aging. The studies analyzed in this systematic review were highly heterogeneous in terms of participants’ characteristics, timing of outcome measurement, and study design. Consequently, assessing the risk of bias is particularly challenging. Selection and confounding bias were the most frequent.

Falls are frequent amongst older adults, it has been reported that one subject out of three after the age of 65 years will fall each year; moreover, age increases injuries caused by the falls [62, 63]. Besides fall-related injuries, falls may generate the fear of falling which leads to reduced independence and decreased physical activity, increasing the development of frailty syndrome [64].

Hyponatremia has been associated with an increased risk of falls in all the analyzed studies, even after correction for confounding factors, except for the study from Hosseini et al. [34]. However, in this study, there was a high risk of selection bias [65, 66]. Moreover, the chosen threshold of 137 mEq/l to define hyponatremia might contribute to equalize the risk of falls between hyponatremic and normonatremic patients according to Tachi et al. [31].

Few studies investigated other conditions associated to increased risk of falling, such as sarcopenia [67], imbalance [68], and gait variability [69]. Only two studies [29, 49] evaluated the incidence of sarcopenia, showing a higher prevalence of this disease in subjects with mild hyponatremia. Gait imbalance was assessed in three studies [9, 29, 49], showing that older subjects with mild hyponatremia had balance impairment.

Although most studies provide evidence of a significant independent association between hyponatremia and falls, almost all the studies were retrospective, so it is not possible to establish a causal role for hyponatremia in determining the risk of falls. Moreover, the majority of the study considered a single time point sodium measurement; thus, information on the duration and persistence of hyponatremia is not available; only two retrospective studies [9, 29] focused on chronic hyponatremia. The study from Ganguli et al. [29] showed a higher incidence of falls only in patients with persistent hyponatremia. These results agree with the results obtained by two prospective studies [4, 34] which did not find any association between hyponatremia and falls and in which a single serum sodium measurement at baseline was used.

The results of the analyzed prospective studies indicate that hyponatremia is a risk factor of falls.

It has been estimated that about 9 million osteoporotic fractures occur every year; thus, osteoporosis accounted for 0.83% of the total chronic noncommunicable disease worldwide and 1.75% of the global burden in terms of incidence of fractures, prevalence of disabled individuals, excess mortality, and disability-adjusted life years (DALYs) in Europe in the year 2000 [70]. Several studies suggested an association between hyponatremia and osteoporotic fractures [4, 10, 29, 36–40, 44, 45, 47]. Hyponatremia has been considered as risk factors for osteoporosis as the skeleton storage about one-third of body sodium and thus can be considered as a reservoir in case of reduction of serum sodium [71]. It has been postulated that in case of severe hyponatremia, sodium is mobilized from bone to maintain blood homeostasis causing matrix resorption and bone loss [7].

Two studies showed that hyponatremia was associated with fragility fractures beyond BMD [19, 43], while four studies [20, 41, 42, 46] showed an increased risk of fragility fractures as a result of a reduction of BMD. On this regard, the study by Afshinnia et al. [41] suggested a role for chronic and not for episodic hyponatremia on the development of osteoporosis, especially in younger patients. According to the authors, age acts as a competing risk factor for osteoporosis by increasing the number of patients with osteoporosis in the normonatremic group due to the old age. This finding is comparable to those of the Rotterdam study [4] that did not find any association between hyponatremia and a lower BMD in older subjects.

In two studies [21, 34], no association between hyponatremia and fragility fractures was found. Some differences in comorbidities between hyponatremic and normonatremic subjects may affect results as confounding factors of the study by Hosseini et al. [34] as previously discussed. Schiara et al. [21] suggested that sodium could be a marker of health status rather than an active player in fracture risk. However, in this study, intravenous fluid infusions or prescribed drugs before admission in cases admitted for femoral fragility fractures such as in the controls admitted for acute myocardial infarction at the emergency department may affect the results. It is noteworthy that the use of antihypertensive medication may influence bone mineral density; in particular, loop diuretics are associated with increased urinary calcium excretion and a consequent increase of parathormone levels that induce an accelerated bone resorption [72]: our results show that this variable was not considered in all the studies as confounding factors [19, 38, 40]. On the other hand, the use of angiotensin-converting enzyme inhibitors has been suggested to increased BMD, thanks to the inhibition of osteoclast activation due to angiotensin I [73]. Additionally, in Hosseini et al. [34], blood urea nitrogen (BUN) was higher in hyponatremic subjects, and increased BUN is associated with a higher protein intake, which, in some studies, has been associated to osteoporosis due to a slight reduction in blood pH and a consequent increase in urinary calcium excretion [74].

All the reviewed studies provide the evidence that hyponatremia is associated with fracture risk; nevertheless, the question whether hyponatremia plays a direct role in increasing the fracture risk and affecting bone density remains to be answered.

Dementia is a syndrome that affects independent living through a decline in memory, cognitive abilities, and behavior and represents a public health priority [75]. The prevalence and incidence of dementia are steadily increasing from 35.6 million worldwide in 2010 [75] to 50 million in 2018, and it will double every 20 years reaching 82 million in 2030 and 152 million in 2050 [76]. It represents the second largest cause of disability for subjects of 70 years and older and the seventh cause of death [77]. The estimated annual global cost of dementia that includes direct medical care, social care, and informal care is actually US $818 billion and is expected to double by 2030 worldwide [77].

Hyponatremia has been studied as risk factor for dementia as neurological symptoms are the main manifestation of severe hyponatremia. However, there are few evidence causally linking chronic hyponatremia to neurological disorders and cognitive impairment. During mild chronic hyponatremia, central nervous system settles an adaptative response involving the loss of osmolytes to prevent swelling and preserve function [78]. However, some of the lost osmolytes are neurotransmitters such as glutamate and taurine [22]; this observation has been summoned to explain the possible derived impairment of cognitive function.

All the studies included in this review evaluated different outcomes and used different methods to assess cognitive abilities; hence, they are highly heterogeneous. Renneboog et al. [9] firstly demonstrated the presence of an attention deficit in mild hyponatremic patients. Gosch et al. [13] as well as Suárez et al. [51] found a worse cognitive performance measured by MMSE in patients with mild to moderate hyponatremia. Despite different study design, retrospective [13] or prospective [51], this two studies found very similar results in MMSE test in hyponatremic patients. Nevertheless, the MMSE score obtained by both these studies is above the cut-off score (23.9/30) that distinguishes between pathological and normal performance [79], thus may not be clinically relevant.

Moreover, the prospective study by Fujisawa et al. [49] showed no alteration in cognitive function in hyponatremic patients despite the use of a full battery of neuropsychological tests to evaluate cognitive performance.

Due to such limited literature and conflicting results, the evidence for an association between hyponatremia and reduced cognitive performance is still lacking. To date, due to the descriptive nature of the studies, the mechanism underlying the association between hyponatremia and cognitive impairment remains unclear. Further studies are required to understand whether cognitive impairment is rather due to the condition causing hyponatremia or if hyponatremia is a causal factor on its own. As discussed above, the main limitation of this review is the type of studies included; due to their observational design and the high heterogeneity, it was not really possible to fully assess the biases and compare the results obtained.

## Conclusions

Referring to Bradford-Hill criteria on causality, biological coherence suggesting a causal effect for hyponatremia was demonstrated for all the outcomes tested. 

Most studies provided consistent evidence for a strong association between hyponatremia and falls, whereas for osteoporotic fractures and cognitive impairment, the studies showed conflicting results. The association with hyponatremia is present, although not very strong, between hyponatremia and fractures. The association between hyponatremia and cognitive impairments has been found to be very weak.

All studies showed that falls, fractures, osteoporosis, and cognitive impairment are multifactorial, and that hyponatremia may not be regarded as a specific cause of the considered diseases.

As regards timing, there are not sufficient evidences to temporally relate hyponatremia with falls, fractures, and cognitive impairment occurrence.

In view of the above considerations, we suggest that hyponatremia may be regarded as a marker of unhealthy aging and a confounder rather than a causal factor or an innocent bystander for falls and fractures. As regards cognitive impairment, the evidence provided until now is not sufficient to explain a real role of hyponatremia that may be regarded rather as an innocent bystander in neurodegeneration.
